# Letter from Springfield, Ill.

**Published:** 1853-10

**Authors:** A. M. French


### Correspondence.

SPRINGFIELD, ILL., *August* 31, 1853.

*Editor of the Dental Register:*

DEAR SIR: We of the borders and backwoods of the great West are accustomed to look to you and your compeers in the practice and teaching of the science and art of the Dentist, in the central and eastern parts of our great country, as to lights set up in regions of light, for illumination and guidance in the pursuit of our devious, ever-changing way, the practice of Dentistry. Accounts of so many new inventions and discoveries are given us by the zealous and enterprising members of the profession, and so many new and often conflicting theories crowd the pages of the dental periodicals, that the most industrious of those whose ambition or talents do not fit to be leaders, must be content to follow very far in the rear.

To put to the test of experience all the new modes of treating peculiar cases as fast as they are made known, is quite impossible, if it could be shown to be desirable.

A certain ubiquitous individual, who figures so largely of late in political and other matters, known as Young America, seems to have taken upon him to give our art a helping hand. His talk on this, as on other subjects, is of progress and advancement. But is it that kind of progress that is generally attended by permanence and stabilility, viz: that which is built on observation and experience? Or does it come of a feverish desire to do something which others have not done, whether the same shall or shall not be of any practical utility. Is it not true that in the practice of the dental, as in that of general medicine and surgery, a host of miscalled improvements are hurried before the profession without having received any adequate trial at the hands of the inventor, very much to the detriment of that individual? Otherwise how do you account for the fact that of so many that encumber the pages of the quarterlies, so few are found of any practical value in the laboratory or operating room? Now, your correspondent wishes to see improvements worthy of the age of steam, but would it be becoming in all the unquestionable sons of genius that adorn and elevate the dental profession, when they feel the pangs of travail and know the hour is come in which a great idea is to be born, to curb their impatience if possible, and wait till the thing be grown a little before recommending to universal use,—or at least submit it as an untried thought.

But I have diverged from the business about which I sat down to write, which was to ask the views of one who exerts much influence over the fraternity on the subject of sending specimens of mechanical dentistry to places of exhibition.

There seems to be much unanimity of opinion on the subject of patents, that it is illiberal and unbecoming an elevated and scientific profession to secure the exclusive use of an invention by letters patent. Can it be thought less so to enter the lists of a public show with the view of bringing oneself into notice by exhibiting the product of one's mechanical skill? Does the Dentist pride himself *chiefly* on what he executes in the laboratory? Or does he do it because superiority is more readily seen and appreciated in this department? Are the conclusions drawn by the public from the comparison of one plate with another just? Every dentist knows very well that it is one thing to fashion, and another to finish a plate of teeth, and quite another to fit them to the human mouth so that they can be worn with comfort, and that they resemble in any considerable degree the human teeth! In the former, the dentist might often be excelled by any jeweler, but for the latter he requires much anatomical and physiological knowledge, cultivated taste, and good judgment, to select and arrange his teeth in a manner becoming the contour of the face and expression of the countenance, as well as sufficient mechanical skill to apply and secure them. It is not unlikely that he who never yet inserted a tooth that answered the purposes of a tooth, shall get the premium and the popular applause, for that too which was fashioned by his neighbor, the jeweler, while he whose works are to-day beautifying the persons, mellowing the voices, and performing perfectly for gastric elaboration the aliment of scores or hundreds of individuals, will be passed unnoticed and unpraised? To me such efforts to get notoriety are akin to the flaming advertising with which some called dentists grace the newspapers, and which I believe is at present discountenanced by most reputable practitioners. It cannot be considered less than a gross deception, because the public do not understand the difference between a piece of dental mechanism for sham and one for use! If those who are ambitious this way would take their patients with them and exhibit at once the wearer and the worn, defective nature finished out by art, I confess that some of my objections would fall—but perhaps this would be incorrect.

Your views upon the subject communicated, either to me privately or to the dental public through your valuable periodical, will be of service to

Your obd't serv't, A. M. French.

[We have ever regarded the exhibition of dental operations *out* of the mouth as of very questionable recommendation. The objections urged by Dr. French are very forcible. The public generally look more to the finish, than what might be really adaptation for service. The latter they cannot judge out of the mouth, and very few, if not dentists, could tell more than the general appearance if seen in the mouth—not one in a thousand could tell where the outer cusp of the front upper bicuspid should strike on the lower teeth. We have never had the time and disposition to make a set of teeth for exhibition.

We have no objection to see dental chairs, spittoons, instruments, and such like, exhibited at our fairs; yet, on such none but dentists should decide as it regards merit. A finely finished chair or instrument might be perfectly useless. Sets of teeth can only be exhibited showing the different styles of work; but because B exhibits a more beautifully finished set of teeth than C, it is no evidence of his superior qualifications as a dentist. This we believe is getting to be the opinion of the public, for they soon learn to discriminate and prefer the recommendation of a tried operation to the mere tinsel which dazzles at a show. We look for the time when public sentiment will correct the evil complained of by Dr. French, just as it has that of advertising. We believe that puffs and paid for editorials, written by the dentist himself, are getting to be considered a want of evidence of skill.—*Ed.*]

